# Intelligent Algorithm-Based Ultrasound for Evaluating the Anesthesia and Nursing Intervention for Elderly Patients with Femoral Intertrochanteric Fractures

**DOI:** 10.1155/2022/3557994

**Published:** 2022-05-24

**Authors:** Zhen Li, Yimei Peng, Liping Zou, Yanfang He

**Affiliations:** Department of Anesthesia Surgery, Changsha Fourth Hospital, Changsha 410006, Hunan, China

## Abstract

This study was aimed to explore the anesthesia, analgesia, and nursing intervention scheme for elderly patients undergoing the operation of intertrochanteric fracture of femur under the guidance of ultrasound optimized by blind deblurring algorithm. Fifty elderly patients undergoing intertrochanteric femoral surgery were randomly enrolled into control group (tracheal intubation intravenous anesthesia + routine nursing) and experimental group (ultrasound-guided nerve block anesthesia + comprehensive nursing based on blind deblurring algorithm), with 25 patients in each group. The effects of anesthesia and recovery were evaluated in the two groups. The results showed that the image evaluation index of blind deblurring algorithm was superior to other algorithms (BM3D, DnCNN, and Red-Net), which improved the quality of ultrasound imaging and was more conducive to intraoperative anesthesia guidance. At the beginning and end of intubation and operation, the fluctuation range of mean arterial pressure (MAP) and heart rate (HR) in the experimental group was lower than that in the control group. The maintenance time of sensory and motor anesthesia block (7.53 ± 1.47 h, 5.45 ± 1.36 h) was longer than that of control group (3.38 ± 1.26 h, 3.02 ± 1.31 h). Visual Analogue Scale/Score (VAS) scores at 6 h, 12 h, and 24 h after surgery were lower than those in the control group. The effective rate of nursing and the incidence of complications (92% and 8%) were better than the control group (80% and 16%), and the difference was statistically significant (*P* < 0.05). In summary, the optimization effect of blind deblurring algorithm was good, which can improve the quality of ultrasound-guided surgery and help in the smooth implementation of surgery. Moreover, nerve block anesthesia and comprehensive nursing were of great value in postoperative analgesia and recovery of patients.

## 1. Introduction

Intertrochanteric fracture of femur is a common orthopedic disease, which refers to fracture occurring from the base of the femoral neck to above the level of the lesser trochanteric [1]. With the increasingly obvious aging trend of China's population, intertrochanteric fracture of femur is gradually common in the elderly population over 65 years old [2]. The elderly suffer from brittle osteoporosis and limited joint activity. If the body suddenly loses balance and falls due to external forces, the affected limb will cause intertrochanteric fracture of femur due to excessive external rotation, internal rotation, or varus [3, 4]. Otherwise, it is caused by the comprehensive injuries of torsion, varus, and hyperextension, such as sudden twisting of the upper body in the fixed state of the lower limbs and collision of the great trochanter with the ground when falling [5]. The clinical symptoms of intertrochanteric fracture of femur are obvious, and patients are usually unable to sit up or walk, with shortening, adduction, and external rotation deformity of lower limbs [6]. Fracture healing is easier after fracture, but coxal varus often occurs, and elderly patients who stay in bed for a long time will cause more complications [7]. Surgery is the main clinical treatment. The elderly are weak, often accompanied by basic diseases, organ function attenuation, and poor tolerance of surgery, so it is necessary to choose safe and appropriate anesthesia. Although traditional endotracheal intubation and intravenous combined anesthesia has a good effect, its application in the operation of intertrochanteric fracture of femur in the elderly may lead to significant fluctuations in hemodynamics, resulting in certain risks. Ultrasound-guided nerve block has the advantages of real-time, accurate, dynamic, and safety, and has been widely used in the treatment of limb anesthesia and pain diseases [8, 9]. It can effectively reduce the influence of anesthetic drugs on the body systems of elderly patients. Guided by ultrasound in local block, the doctors can clearly see the nerve structure and the blood vessels, muscles, bones, and visceral structures around the nerve. In the process of needle insertion, real-time images of needle movement can be provided to better approach the target structure [10–12], which is conducive to the smooth implementation of surgery.

However, there are some uncontrollable factors such as breathing movement, blood flow, and mechanical interference in ultrasonic monitoring, often resulting in blurred image or speckle noise, thereby affecting the intraoperative guidance effect. In recent years, medical image deblurring algorithms have been widely used to deal with the problem of speckle noise or blur in images. Blind deblurring refers to the fact that existing fuzzy ultrasonic images are known, while unknown high-quality images and fuzzy nuclei should be estimated by extracting the observation data of fuzzy images and existing prior knowledge [13, 14]. In ultrasound imaging, blind deblurring is more applicable than non-blind deblurring and has great application value [15]. Elderly patients are weak in constitution, need to stay in bed for a long time during treatment, and are prone to postoperative complications. Therefore, it is necessary to provide patients with scientific and effective nursing intervention methods to improve their postoperative quality of life and rehabilitation effect [16].

In this study, 50 elderly patients with intertrochanteric fracture of femur were selected as the research object, and different anesthesia methods and nursing interventions were adopted to explore the anesthesia and analgesia effect and nursing effect of ultrasound-guided nerve block combined with comprehensive nursing based on blind deblurring algorithm in the operation of intertrochanteric fracture of femur, to provide an effective and feasible program for the treatment and postoperative nursing of intertrochanteric fracture of femur in elderly patients.

## 2. Materials and Methods

### 2.1. Research Objects

A total of 50 patients with intertrochanteric fracture of femur, including 30 males and 20 females, who were admitted to the hospital from August 2019 to August 2021, were selected. The age range was 60–78 years, with an average age of 65.3 ± 3.2 years. All patients underwent surgery for intertrochanteric fracture of femur, and were randomly divided into control group and experimental group (25 cases each). Patients in the control group received endotracheal intubation intravenous anesthesia combined with routine nursing intervention. Patients in the experimental group took blind deblurring algorithm-based ultrasound-guided nerve block anesthesia combined with comprehensive nursing intervention. During the study period, no harm was caused to patients. The subjects agreed to sign informed consent with the consent of their family members and this study was approved by ethics committee of hospital.

  Inclusion criteria: (i) Intertrochanteric fracture of femur was confirmed through relevant clinical examination; (ii) Closed fracture; (iii) Age ≥60 years; (iv) Rated level II–IV by the *American Society of Anesthesiologists* (ASA).
  Exclusion criteria: (i) Patients with open, pathological, and old fractures; (ii) Patients with serious organic diseases; (iii) Patients with surgical contraindications; (iv) Patients with incomplete clinical data; (v) Patients with poor compliance and who did not cooperate with the experiment.

### 2.2. Blind Deblurring Algorithm

In addition to feature information, medical images also contain most of the invalid information, which is called sparsity. Studies suggested that the *L*0 norm of the image has a good denoising effect. Therefore, in the blind deblurring of this experiment, the blind deblurring mainly relied on the prior information of the image, so the *L*0 norm of the ultrasound image was used to represent its sparsity, and it was also used as the prior information. Assuming an ultrasound image as *M*, the dark channel of the image can be defined as the following equation.

(1) $$\begin{array}{c} A\left(M\right)\left(u\right)=\underset{v\in \phi \left(u\right)}{\min}\left(\underset{n\in \left\{d,e,f\right\}}{{\mathrm{minM}}^{n}\left(v\right)}\right). \end{array}$$

In equation (1), *u* and *v* represent the pixels of ultrasound imaging, *ϕ*(*u*) represents the image module with *u* as the center, and *M*^*n*^ represents the *N*th color channel. When *M* is a grayscale image, there is $\underset{n\in \left\{d,e,f\right\}}{\min}M^{n}\left(v\right)=M\left(v\right)$. Then, the secondary sparse dark channel of *M* can be defined as follows.

(2) $$\begin{array}{c} SA\left(M\right)\left(u\right)=\underset{v\in \phi \left(u\right)}{\min}A\left(M\right)\left(v\right). \end{array}$$

Likewise, the bright channel of ultrasound imaging *M* is defined as follows.

(3) $$\begin{array}{c} Z\left(M\right)\left(u\right)=\underset{v\in \phi \left(u\right)}{\max}\left(\underset{N\in \left\{d,e,f\right\}}{{\mathrm{maxM}}^{N}\left(v\right)}\right). \end{array}$$

In equation (3), *u* and *v* represent the pixels of ultrasound imaging, *ϕ*(*u*) represents the image module with *u* as the center, and *M*^*N*^ represents the *N*th color channel. When *M* is a grayscale image, there is $\underset{N\in \left\{d,e,f\right\}}{\max}M^{N}\left(v\right)=M\left(v\right)$. Then, the secondary sparse dark channel of *M* can be defined as follows.

(4) $$\begin{array}{c} SZ\left(M\right)\left(u\right)=\underset{v\in \phi \left(u\right)}{\max}Z\left(M\right)\left(v\right). \end{array}$$

However, in medical images, both channels have certain limitations, so a secondary sparse extreme channel prior blind deblurring model is proposed, which is defined as follows.

(5) $$\begin{array}{c} \left\{\hat{J},\hat{h}\right\}=\arg\underset{J,\text{h}}{\min}{\left\|J⊗h-Z\right\|}_{2}^{2}=\varphi q\left(h\right)+\vartheta q\left(J\right). \end{array}$$

In equation (5), *q*(*h*) represents the prior information of the fuzzy kernel, and *q*(*J*) represents the prior information of the secondary sparse extreme channel, both *φ* and *ϑ* are weight parameters. For the estimation of a clear and high-quality ultrasound image *M*, it can be expressed as follows.

(6) $$\begin{array}{c} \hat{M}=\arg\underset{M}{\min}{\left\|J⊗h-Z\right\|}_{2}^{2}+\theta {\left\|SA\left(M\right)-q\right\|}_{2}^{2}+\zeta {\left\|1-SZ\left(M\right)-q\right\|}_{2}^{2}. \end{array}$$

Peak signal-to-noise ratio (PSNR) and structural similarity (SSIM) are used to evaluate the effect of ultrasonic images. PSNR is a measure of image quality, often expressed in decibels (dB) units. To calculate PSNR, the calculation of mean-square error (MSE) should be calculated first. It is supposed that two *m* × *n* monochromatic images are *I* and *K*. If the noise of one is similar to that of the other, the MSE is defined as follows.

(7) $$\begin{array}{c} MSE=\frac{1}{mn}{\sum_{i=0}^{m-1}\sum_{j=0}^{n-1}\left[I\left(i,j\right)-K\left(i,j\right)\right]}^{2}. \end{array}$$

MSE is a common loss function, while PSNR is obtained through MSE. The equation is expressed as follows.

(8) $$\begin{array}{c} \text{PSNR}=10\cdot \;\log_{10}\left(\frac{{\text{MAX}}_{I}^{2}}{\text{MSE}}\right)=20\cdot \;\log_{10}\left(\frac{{\text{MAX}}_{I}}{\sqrt{\text{MSE}}}\right). \end{array}$$

PSNR higher than 40 dB indicates excellent image quality, that is, very close to the original image. A range of 30–40 dB usually indicates that the image quality is good (that is, the distortion can be detected but acceptable), and a range of 20–30 dB indicates that the image quality is poor. PSNR below 20 dB indicates that the image is unacceptable.

SSIM is an indicator to measure the similarity of two images and is often used to evaluate the image quality. The input of SSIM is two images. Assuming that the input two images are *I* and *Y*, respectively, the equation is expressed as follows.

(9) $$\begin{array}{c} \text{SSIM}\left(x,y\right)={\left[l\left(x,y\right)\right]}^{\alpha }{\left[c\left(x,y\right)\right]}^{\beta }{\left[s\left(x,y\right)\right]}^{\gamma }, \end{array}$$

*l*(*x*, *y*) is brightness comparison, *c*(*x*, *y*) is contrast comparison, and *s*(*x*, *y*) is structure comparison. SSIM is a number between 0 and 1, the larger the value is, the smaller the gap between the output image and the undistorted image is, that is, the better the image quality is.

### 2.3. Anesthesia Methods

The patients were deprived of water for 4 h and fasted for 6–8 h before surgery. Intravenous channels were established before patient's entering the room, and oxygen was given by routine mask after entering the room. Heart rate (HR) and mean arterial pressure (MAP) were carefully detected. Patients in the control group received endotracheal intubation intravenous combined anesthesia, which was induced by anesthesia with 2 mg·kg^−1^ propofol and 0.08 mg·kg^−1^ vecuronium before surgery. Endotracheal intubation and anesthesia machine were connected. Intraoperatively, 2–3 mg·kg^−1^·h^−1^ propofol and 0.2–0.25 *μ*g·kg^−1^·min^−1^ vecuronium were administered to maintain anesthesia. Drug administration was stopped five minutes after the end of surgery, and the catheter was removed after the patient was awake.

The patients in the experimental group received ultrasound-guided nerve block anesthesia based on blind deblurring algorithm, and the anesthesia induction and maintenance were the same as the control group. The patient was placed in supine position, and the femoral artery pulsation was palpated at the midpoint of the inguinal ligament with a conventional disinfection towel. After probing of the position of the femoral vein, femoral artery, and femoral nerve by the ultrasound system with a 2–5 MHz probe, 0.33%–0.5% ropivacaine hydrochloride was injected perinatal to the femoral nerve under ultrasound monitoring.

### 2.4. Proximal Femoral Nail Antirotation Internal Fixation

The affected limb was pulled through the bedside C-arm fluoroscopy, and the side of the fracture was closed for reduction. After the reduction was confirmed, routine disinfection of the cloth was performed. A longitudinal incision was made above the greater trochanter of the femur, with successive incisions exposing the tip of the greater trochanter. An anterior opening was made with a mouthpiece, and a guide wire was inserted to confirm placement in the medullary cavity, thereby dilating the pulp proximal and inserting the intramedullary needle main nail. After installing the sights, a Kirschner wire was placed in the neck of the femur, thereby drilling and tapping after confirming the appropriate position. After the screw blade compression screw was inserted, the distal sight was installed, and the distal locking nail was inserted. After observation and reduction, the incision was closed. Postoperative antibiotics were given to prevent infection and low molecular weight heparin calcium to prevent deep vein thrombosis.

### 2.5. Nursing Interventions

The patients in the control group were given routine care, including health knowledge education for the patients, good ward environment care, and close observation of the patients' conditions.

On this basis, patients in the experimental group took comprehensive nursing, including summarizing and analyzing the surgical nursing problems of patients, as well as referring to relevant documents to sort out the correct treatment methods. According to the patient's own situation for psychological counseling, it was required to reduce their tension, anxiety, fear, and other negative emotions, and patiently answer the questions raised by patients and their families. The patients should be assisted with limb activities and rehabilitation training, to promote blood circulation to reduce the pain of patients. Five hours after the operation, the affected limb should be raised, and the ECG, blood pressure, and other indicators should be observed on time, as well as whether there was bleeding infection at the incision. The temperature and skin color of the affected limb were recorded in detail. It was also required to actively assist patients to turn over and buckle back, and massage affected limbs to promote their blood circulation. The wound dressing was changed in time to prevent wound infection and other complications.

### 2.6. Observation Indicators

The hemodynamic indexes HR and MAP of two groups were observed and recorded at the time points before surgery (*T*0), at intubation (*T*1), at the beginning of surgery (*T*2), and at the end of surgery (*T*3). The maintenance time of anesthesia block in two groups was observed. Visual Analogue Scale/Score (VAS) was used to assess the pain degree of patients. Zero was no pain and ten was severe pain. The postoperative recovery of the two groups was observed. Effective: imaging examination showed that the fracture was fully healed, the hip flexion and extension activities were normal, and no serious complications occurred. Significant effective: the fracture was basically healed, and the hip joint activity function was basically normal. Ineffective: no healing was observed at the fracture site, limited hip movement, and obvious pain and limb deformity occurred. The incidence of complications in two groups was observed and recorded.

### 2.7. Statistical Analysis

SPSS 24.0 was employed for data statistics and analysis. Mean ± standard deviation ($\bar{x}$ ± *s*) was how measurement data were expressed, and the *t*-test was used for measurement data conformed to normal distribution. Count data was tested by *χ*^2^ test. The difference was statistically considerable with *P* < 0.05.

## 3. Results

### 3.1. Blind Deblurring Algorithm-Based Ultrasound Guidance

**Figure 1:**
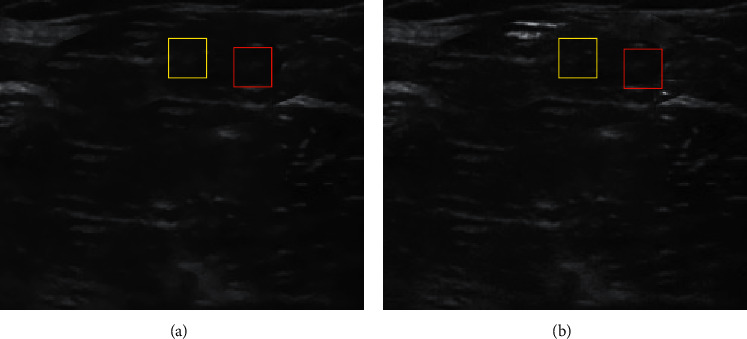
Ultrasound guidance based on blind deblurring algorithm. (a) was the initial ultrasound-guided imaging, and (b) was the ultrasound-guided imaging after blind deblurring algorithm optimization.

Figure 1 shows that the ultrasound imaging based on blind deblurring algorithm was relatively clearer, with less speckle noise and higher image quality. In Figure 1, the yellow area is the spread of the anesthetic and the red area is the lateral femoral nerve. The proposed algorithm was compared with other deblurring algorithms (BM3D, DnCNN, and Red-Net) (Table 1), and the proposed algorithm achieved the highest average PSNR and SSIM, and the deblurring effect was relatively ideal.

### 3.2. Comparison of Basic Clinical Data of Two Groups of Patients

Table 2 shows that there was no significant difference in basic clinical data such as age, sex ratio, body mass index (BMI), and cause of injury between the control group and the experimental group (*P* > 0.05), which were comparable.

### 3.3. Comparison of Hemodynamics between Two Groups of Patients at Different Time Points

**Figure 2:**
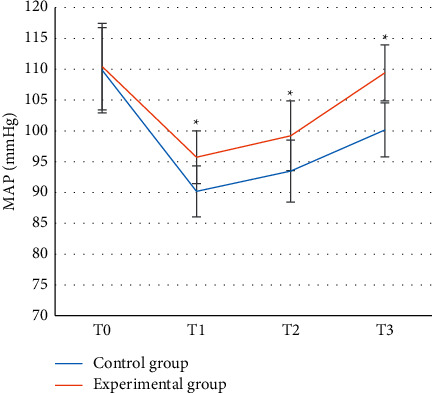
MAP comparison of two groups of patients at different time points. ^*∗*^represented that the fluctuation range of the experimental group was significantly different from that of the control group (*P* < 0.05).

**Figure 3:**
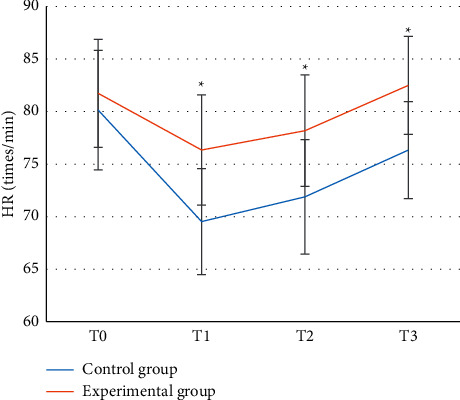
HR comparison of two groups of patients at different time points. ^*∗*^represented that the fluctuation range of the experimental group was significantly different from that of the control group (*P* < 0.05).

Figures 2 and 3 show that the difference in MAP and HR between the control group and the experimental group was not statistically significant at *T*0 (*P* > 0.05), while at *T*1–*T*3, the fluctuation range of MAP and HR in the experimental group was lower than that of the control group, and the difference was statistically significant (*P* < 0.05).

### 3.4. Comparison of Anesthesia Block Maintenance Time between Two Groups of Patients

**Figure 4:**
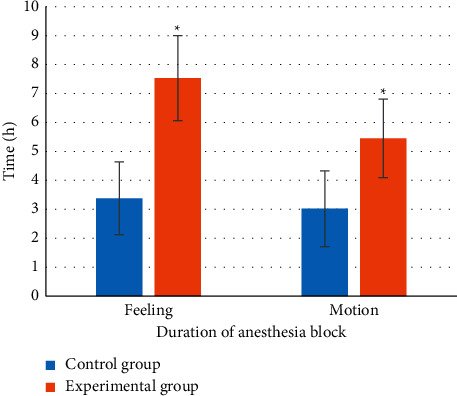
Comparison of anesthesia block maintenance time between two groups of patients. ^*∗*^represented that the maintenance time of sensory and motor anesthesia block in the experimental group was significantly different from that in the control group (*P* < 0.05).

Figure 4 shows that the duration of sensory and motor anesthesia block in the experimental group was longer than that in the control group, and the difference was statistically significant (*P* < 0.05).

### 3.5. Comparison of Postoperative Pain in Two Groups of Patients

**Figure 5:**
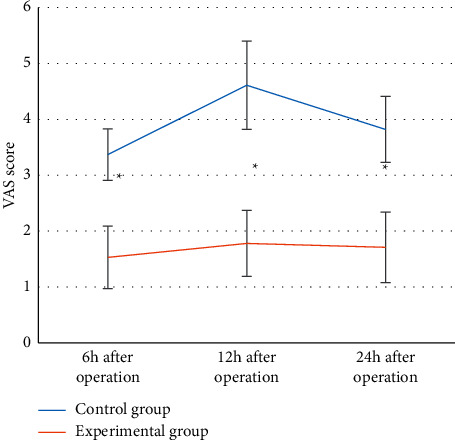
Comparison of postoperative pain levels between the two groups of patients. ^*∗*^represented that the postoperative pain degree of the experimental group was significantly different from that of the control group (*P* < 0.05).

In Figure 5, the VAS scores of the experimental group were lower than those of the control group at 6 h, 12 h, and 24 h after surgery, and the difference was statistically significant (*P* < 0.05).

### 3.6. Comparison of Nursing Effect of Two Groups of Patients

**Figure 6:**
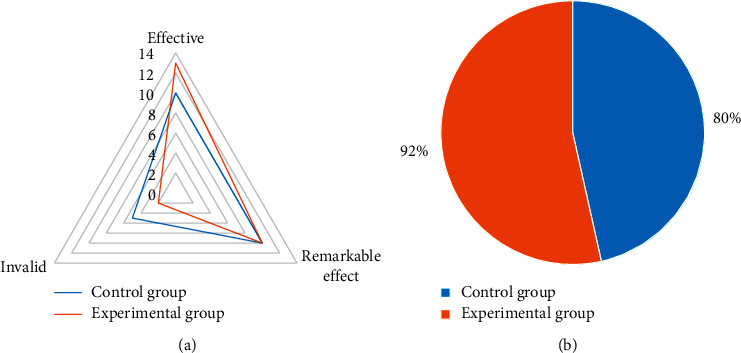
Comparison of nursing effect of two groups of patients. (a) comparison of nursing effect of two groups of patients, (b) comparison of total effective rate of nursing of two groups of patients.

In Figure 6, 10 cases of nursing effect were effective, 10 cases were markedly effective, and 5 cases were ineffective in the control group, and the total effective rate of nursing was 80%. In the experimental group, the nursing effect was effective in 13 cases, markedly effective in 10 cases, and ineffective in 2 cases, and the total nursing effective rate was 92%. The nursing effective rate of the experimental group was significantly higher than that of the control group (*P* < 0.05).

### 3.7. Comparison of Postoperative Complication Rates between the Two Groups of Patients

**Figure 7:**
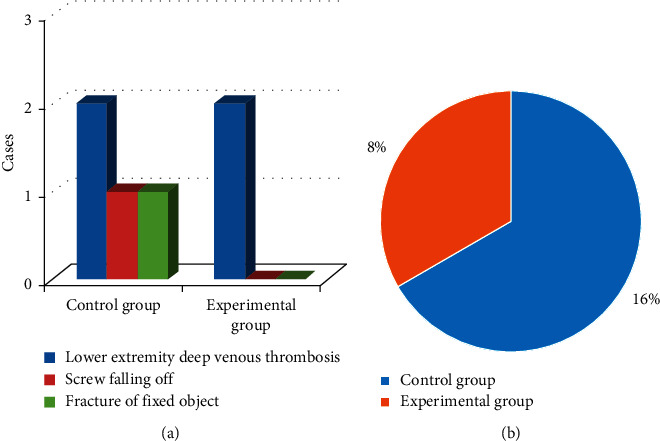
Comparison of postoperative complication rates between the two groups of patients. (a): the incidence of postoperative complications in the two groups of patients; (b): the comparison of the incidence of postoperative complications in the two groups of patients.

In Figure 7, there were 2 cases of lower extremity deep venous thrombosis, 1 case of screw fell off, and 1 case of fixation rupture in the control group, and the complication rate was 16%. There were 2 cases of lower extremity deep vein thrombosis in the experimental group, and the complication rate was 8%. The incidence of complications in the experimental group was significantly lower than that in the control group, and the difference was statistically significant (*P* < 0.05).

## 4. Discussion

Intertrochanteric fracture of femur refers to the fracture from the base of the femoral neck to above the level of the lesser trochanteric, which is a common disease in the elderly. With the aging of society, the incidence of intertrochanteric fracture of femur has increased significantly [17, 18]. Intertrochanteric fracture of femur in the elderly is often comminuted fracture, accompanied by osteoporosis, and often complicated with a variety of complications. Traditional internal fixation can effectively fix multiple fracture blocks, achieve the fixation strength required for fracture end healing, and maintain a stable angle, which is an ideal surgical method for the treatment of senile intertrochanteric fracture [19]. However, there are degenerative changes in various organs of the elderly patients, with reduced or atrophic functional cells, low storage capacity, and compensatory stress ability. Their tolerance of anesthesia and operation is not strong, so there are many risks in anesthesia operation. Moreover, since patients need to stay in bed during the treatment, the possibility of complications is high, and the elderly will have fear and anxiety about anesthesia and surgery. If the nursing is improper, it will affect the postoperative rehabilitation effect, so it is very necessary to choose the appropriate anesthesia and nursing mode.

In this experiment, patients in the control group were given endotracheal intubation intravenous combined anesthesia, and patients in the experimental group were given ultrasound-guided nerve block anesthesia based on blind deblurring algorithm. Hemodynamics and maintenance time of anesthesia block were observed and recorded before operation (*T*0), at intubation (*T*1), at the beginning of operation (*T*2), and at the end of operation (*T*3). The results showed that the blind deblurring algorithm can improve the quality of ultrasound imaging and conduct anesthesia guidance more effectively. At *T*1–*T*3, the fluctuation range of MAP and HR in the experimental group was lower than that in the control group, and the maintenance time of sensory and motor anesthesia block in the experimental group was longer than that in the control group. Li et al. [20] also demonstrated similar verification, indicating that ultrasound-guided nerve block anesthesia based on blind deblurring algorithm can maintain intraoperative hemodynamic stability and prolong the maintenance time of anesthesia block. This was because ultrasound-guided nerve block was used to explore and define the anatomy of the perineural block, in which a reasonable choice of puncture path can be made to avoid the surrounding organs and blood vessels, resulting in a higher puncture success rate and a better effect of anesthesia. At the same time, the needle tip was close to the nerve, the anesthetic drug was fully infiltrated into each nerve bundle, and the anesthetic time was subsequently prolonged. Moreover, this blocking effect was only for unilateral nerve, and had little effect on sympathetic nerve, so intraoperative hemodynamics was relatively stable. The maintenance time of sensory and motor anesthesia block in experimental group was longer than that in control group. These results indicate that ultrasound-guided nerve block anesthesia based on blind deblurring algorithm can better maintain intraoperative hemodynamic stability and prolong the maintenance time of anesthesia block. Then, VAS was used to evaluate the pain degree of patients in the two groups, and the results showed that VAS scores of patients in the experimental group were lower than those in the control group at 6 h, 12 h, and 24 h after surgery, with statistically significant differences (*P* < 0.05). This was consistent with what Omoregbe et al. [21] stated that ultrasound-guided nerve block anesthesia had good anesthesia and analgesic effects in surgery. By observing the postoperative nursing efficiency and complication rate of the two groups, it was known that the total nursing efficiency and complication rate of the experimental group with comprehensive nursing were better than the control group with conventional nursing. It showed that comprehensive nursing can significantly improve the nursing effect of patients with intertrochanteric fracture of femur and reduce the incidence of complications. Fan et al. [22] also pointed out that comprehensive nursing was a nursing intervention model emerging in recent years, which can improve the nursing effect and satisfaction of patients by combining previous nursing experience and effectively reduce the occurrence of postoperative complications.

## 5. Conclusion

Patients in the control group and the experimental group were given different anesthesia methods and nursing interventions. The experiment showed that the ultrasound image was clearer under blind deblurring algorithm optimization, and there was no excess speckle noise. Ultrasound-guided nerve block anesthesia based on blind deblurring algorithm can stabilize hemodynamics of patients with long anesthesia block time and less pain. Comprehensive nursing can improve nursing efficiency and reduce the incidence of complications. However, the deficiency of this study is that fewer samples were collected, so more clinical data need to be collected for research. In addition, VAS scoring is subjective to some extent, and the statistical results may be one-sided and limited. In conclusion, ultrasound-guided nerve block anesthesia and comprehensive nursing based on blind deblurring algorithm play a positive role in the operation of patients with intertrochanteric fracture of femur, and are worthy of clinical application.

## Figures and Tables

**Table 1:** Effect comparison of different deblurring algorithms.

Deblurring algorithms	PSNR (dB)	SSIM (dB)
BM3D	25.462	0.753
DnCNN	31.479	0.697
Red-net	28.368	0.787
The proposed algorithm	36.872	0.895

**Table 2:** Comparison of basic clinical data of the two groups of patients.

Clinical information	Control group (25 cases)	Experimental group (25 cases)
Age (years old)	65.1 ± 3.6	65.4 ± 3.3
Gender (male/female)	14/11	16/9
BMI (kg/m^2^)	20.14 ± 0.41	21.08 ± 0.45
Cause of injury—fall (case)	18	17
Cause of injury—fall from height (case)	5	5
Cause of injury—car accident (case)	2	3

## Data Availability

The data used to support the findings of this study are available from the corresponding author upon request.

## References

[1] Karakus O., Ozdemir G., Karaca S., Cetin M., Saygi B. (2018). The relationship between the type of unstable intertrochanteric femur fracture and mobility in the elderly. *Journal of Orthopaedic Surgery and Research*.

[2] Lv Z., Qiao L. (2020). Analysis of healthcare big data. *Future Generation Computer Systems*.

[3] Zhao Z., Chen J., Li X., Liu L., Wei W., Wang G. (2020). Progress on reconstruction of proximal femur in the hemiarthroplasty for intertrochanteric fracture with distal fixated long stem. *Zhongguo Xiu Fu Chong Jian Wai Ke Za Zhi*.

[4] Lin J. A., Cui H. D., Hong Y., Lyu S. J. (2021). Application of tranexamic acid in the treatment of intertrochanteric fracture of femur. *Zhong Guo Gu Shang*.

[5] Mascoe J. E., Herickhoff P. K. (2021). Conservative treatment of a nondisplaced intertrochanteric femur fracture: a case report and review of the literature. *The Iowa Orthopaedic Journal*.

[6] Bedrettin A., Sahin F., Yucel M. O. (2022). Treatment of intertrochanteric femur fracture with closed external fixation in high-risk geriatric patients: can it be the most reliable method that reduces mortality to minimum compared to proximal femoral nail and hemiarthroplasty?. *Medicine*.

[7] Ding Q., Wang C. L., Wang P. F., Zuo C. H., Xie W., Sun L. Y. (2020). Treatment of intertrochanteric fracture of femur with closed reduction of proximal femoral anti rotation intramedullary nail in supine position. *Zhong Guo Gu Shang*.

[8] Kim D. H., Lee M. S., Lee S., Yoon S. H., Shin J. W., Choi S. S. (2019). A prospective randomized comparison of the efficacy of ultrasound- vs fluoroscopy-guided genicular nerve block for chronic knee osteoarthritis. *Pain Physician*.

[9] Kang C., Hwang D.-S., Song J.-H., Lee G.-S. J.-K. S.-J. J.-H. B.-K. (2021). Clinical analyses of ultrasound-guided nerve block in lower-extremity surgery: a retrospective study. *Journal of Orthopaedic Surgery*.

[10] Lee M. G., Choi S. U., Lim J. K. (2020). Ultrasound‐guided sciatic nerve block at the midthigh level in a porcine model: a descriptive study. *Veterinary Medicine and Science*.

[11] Li Y., Zhang Q., Wang Y. (2021). Ultrasound-guided single popliteal sciatic nerve block is an effective postoperative analgesia strategy for calcaneal fracture: a randomized clinical trial. *BMC Musculoskeletal Disorders*.

[12] Liu Y., Cheng L. (2021). Ultrasound images guided under deep learning in the anesthesia effect of the regional nerve block on scapular fracture surgery. *J Healthc Eng*.

[13] Xie S., Yu Z., Lv Z. (2021). Multi-disease prediction based on deep learning: a survey. *Computer Modeling in Engineering and Sciences*.

[14] Huang L., Xia Y., Ye T. (2021). Effective blind image deblurring using matrix-variable optimization. *IEEE Transactions on Image Processing*.

[15] Li Y., Zhao J., Lv Z., Li J. (2021). Medical image fusion method by deep learning. *International Journal of Cognitive Computing in Engineering*.

[16] Xu Y., Wang R. Y., Zhao Y. H. (2021). Effects of perioperative comprehensive nursing based on risk prevention for patients with intracranial aneurysm. *International Journal of Clinical Practice*.

[17] Hou Y., Yao Q., Zhang G., Ding L. (2018). A clinical study on the relationship of the tail femur distance and the lag screw migration or cutting-out after the third generation of Gamma nail fixation of intertrochanteric fracture. *Zhongguo Xiu Fu Chong Jian Wai Ke Za Zhi*.

[18] Kempegowda H., Richard R., Borade A., Tawari A. J. M. A. E. N. V. R. K. F. A. N. D. S. (2017). Obesity is associated with high perioperative complications among surgically treated intertrochanteric fracture of the femur. *Journal of Orthopaedic Trauma*.

[19] Li H., Zhang W., Yan J. (2018). Greater trochanter of the femur (GTF) vs. proximal femoral nail anti-rotation (PFNA) for unstable intertrochanteric femoral fracture. *European Review for Medical and Pharmacological Sciences*.

[20] Li Z., Zhao L., Wang W., Zheng L. (2021). Application of intelligent ultrasound in real-time monitoring of postoperative analgesic nerve block. *Contrast Media and Molecular Imaging*.

[21] Omoregbe O. R., Idehen H. O., Imarengiaye C. O. (2020). Supraclavicular brachial plexus block for upper limb fracture fixation:A comparison of nerve stimulation, ultrasound-guided technique and a combination of both techniques. *West African Journal of Medicine*.

[22] Fan D., Han L., Qu W. (2019). Comprehensive nursing based on feedforward control and postoperative FMA and SF-36 levels in femoral intertrochanteric fracture. *Journal of Musculoskeletal and Neuronal Interactions*.

